# Dl-3-n-Butylphthalide Alleviates Behavioral and Cognitive Symptoms Via Modulating Mitochondrial Dynamics in the A53T-α-Synuclein Mouse Model of Parkinson’s Disease

**DOI:** 10.3389/fnins.2021.647266

**Published:** 2021-05-28

**Authors:** Huiying Li, Hongquan Wang, Ling Zhang, Manshi Wang, Yanfeng Li

**Affiliations:** ^1^Department of Neurology, Beijing Aerospace General Hospital, Beijing, China; ^2^Department of Neurology, Aerospace Center Hospital, Peking University Aerospace School of Clinical Medicine, Beijing, China; ^3^Key Laboratory of Human Disease Comparative Medicine, Chinese Ministry of Health, Institute of Laboratory Animal Science, Chinese Academy of Medical Sciences, Beijing, China; ^4^Key Laboratory of Human Diseases Animal Models, State Administration of Traditional Chinese Medicine, Peking Union Medicine College, Beijing, China; ^5^Department of Neurology, Peking Union Medical College Hospital, Beijing, China

**Keywords:** Dl-3-n-butylphthalide, α-synuclein, mitochondrial dynamics, Parkinson’s disease, behavior deficits, cognitive symptoms

## Abstract

**Background:**

Aggregation and neurotoxicity of the presynaptic protein α-synuclein and the progressive loss of nigral dopaminergic neurons are believed to be the key hallmarks of Parkinson’s disease (PD). A53T mutant α-synuclein causes early onset PD and more severe manifestations. A growing body of evidence shows that misfolding or deposition of α-synuclein is linked to the maintenance of mitochondrial dynamics, which has been proven to play an important role in the pathogenesis of PD. It has been observed that Dl-3-n-butylphthalide (NBP) may be safe and effective in improving the non-tremor-dominant PD. However, the potential mechanism remains unclear. This study aimed to investigate whether NBP could decrease the loss of dopaminergic neurons and α-synuclein deposition and explore its possible neuroprotective mechanisms.

**Methods:**

A total of 20 twelve-month-old human A53T α-synuclein transgenic mice and 10 matched adult C57BL/6 mice were included in the study; 10 adult C57BL/6 mice were selected as the control group and administered with saline (0.2 ml daily for 14 days); 20 human A53T α-synuclein transgenic mice were randomly divided into A53T group (treated in the same manner as in the control group) and A53T + NBP group (treated with NBP 0.2 ml daily for 14 days). Several markers of mitochondrial fission and fusion and mitophagy were determined, and the behavioral, olfactory, and cognitive symptoms were assessed as well.

**Results:**

In the present study, it was observed that the A53T-α-synuclein PD mice exhibited anxiety-like behavioral disturbance, impairment of coordination ability, memory deficits, and olfactory dysfunction, loss of dopaminergic neurons, and α-synuclein accumulation. Meanwhile, the mitofusin 1 expression was significantly decreased, and the mitochondrial number and dynamin-related protein 1, Parkin, and LC3 levels were increased. The detected levels of all markers were reversed by NBP treatment, and the mitochondrial morphology was partially recovered.

**Conclusion:**

In the present study, a valuable neuropharmacological role of NBP has been established in the A53T-α-synuclein PD mouse model. Possible neuroprotective mechanisms might be that NBP is involved in the maintenance of mitochondrial dynamics including mitochondrial fission and fusion and clearance of damaged mitochondria. It is essential to perform further experiments to shed light on the precise mechanisms of NBP on mitochondrial homeostasis.

## Introduction

Parkinson’s disease (PD) is the second most common neurodegenerative disorder in the world, affecting up to 2% of the population over 65 years old (Ray et al., 2018). Besides the typical clinical motor symptoms, behavioral and cognitive symptoms and olfactory dysfunction are highly prevalent in PD. These non-motor symptoms may occur even in the prodromal stages of the disease, worsen with disease progression, and surpass motor symptoms as the major factors affecting the patient’s quality of life and the caregiver’s burden (Martinez-Martin et al., 2015; Prakash et al., 2016). In the early stage of PD, dopamine replacement therapy and deep brain stimulation can improve the levodopa-responsive symptoms and reduce hyperdopaminergic behaviors but cannot slow down the progression of the disease (Bronstein et al., 2011; Olanow, 2019). With the progression of PD, current treatments for motor symptoms are less effective than those in the early stage, and antiparkinsonian drugs can potentially induce side effects and adverse drug reactions (Carrarini et al., 2019). As one of the adverse effects of prolonged use of dopaminergic drugs, the behavioral symptoms may reappear. Cholinergic deficits may particularly cause dementia in PD patients (Rektorova, 2019). Therefore, it is urgent to identify the promising neuroprotective or disease-modifying drugs to alleviate these non-motor symptoms, slow down the progression of PD, or tackle the cause of the disease.

Although the aggregation and neurotoxicity of the presynaptic protein α-synuclein and the progressive loss of nigral dopaminergic neurons are believed to be the key hallmarks of PD, the exact pathophysiological mechanisms of occurrence and progression in PD remain unclear. α-Synuclein A53T mutation causes early onset PD and more severe manifestations (Puschmann et al., 2009). Several lines of evidence show that misfolding or aggregation of α-synuclein is linked to the maintenance of mitochondrial dynamics including mitochondrial fusion–fission, transport, and mitophagy, which has been proven to play an important role in the pathogenesis of PD (Pozo Devoto and Falzone, 2017; Ordonez et al., 2018; Vicario et al., 2018), and this effect is particularly prominent in α-synuclein A53T mutation (Xie and Chung, 2012). Several studies have suggested that modulating mitochondrial dynamics might be a potential therapeutic strategy for the treatment of PD (Oettinghaus et al., 2012; Hijaz and Volpicelli-Daley, 2020; Park et al., 2020).

It has been observed that Dl-3-n-butylphthalide (NBP), which is extracted from the seeds of *Apium graveolens* in 1978, could be safe and effective in improving the non-motor symptoms and sleep disorders of patients with non-tremor-dominant PD (Zhou et al., 2019). It was reported in a recent study that NBP exerts neuroprotective effects against oxidative damage and mitochondrial dysfunction through its antioxidant property via an autophagy mechanism in a 1-methyl-4-phenylpyridinium^+^-induced cellular model of PD (Huang et al., 2010). Despite this evidence, it is unclear whether NBP could play a beneficial role in human A53T α-synuclein transgenic PD mouse, a commonly used PD mice model given rise to dominant early onset and non-motor manifestations, and speculate that the potential mechanism might be linked to mitochondrial dynamics.

The present study was designed to investigate the possible neuroprotective role of NBP in a human A53T α-synuclein transgenic mouse model of PD to further explore the potential mechanism on mitochondrial dynamics. To elucidate the possible mitochondrial mechanism involved in NBP-induced neuroprotection in human A53T α-synuclein transgenic mouse model of PD, the effects of NBP on behavior, cognition, olfactory function, α-synuclein levels, and the number of tyrosine hydroxylase-positive cells in the corpus striatum and substantia nigra were examined, and mitochondrial proteins including fission, fusion, and mitophagy were monitored as well in model mice.

## Materials and Methods

### Reagents

The following reagents were used: anti-DRP1 #8570 (Cell Signaling Technology, United States), anti-MFN1 #12186-AP (Proteintech Group, United States), anti-Parkin #2132s (Cell Signaling Technology, United States), anti-LC3 #3868s (Cell Signaling Technology, United States), tyrosine hydroxylase (TH) #EP1532Y (Abcam, United Kingdom), α-synuclein #D37A6 (Cell Signaling Technology, United States), and NBP (25 mg/5 ml) (CSPC Pharmaceutical Co., Ltd., China).

### Animals and Treatment

A total of 10 adult C57BL/6 mice and 20 A53T α-synuclein transgenic mice expressing mutant human A53T α-synuclein, 12 months old, weighing 25–35 g, with a gender ratio of 1:1 [Institute of Laboratory Animal Science, Chinese Academy of Medical Sciences, Beijing, China; No. SCXK (Jing) 2016-0006] were raised in a specific pathogen-free grade experimental animal facility in Beijing Weitonglihua Company. The temperature of the room with a mechanical ventilation system was set at 18–28°C, with 40–70% relative humidity, 150–300 lx luminance under a 12 h/12 h light/dark cycle, and food and water available *ad libitum*.

The total mice were divided into three groups. Ten adult C57BL/6 mice were selected as the control group (with a gender ratio of 1:1) and intraperitoneally injected with saline 0.2 ml daily for 14 days. Twenty A53T α-synuclein transgenic mice were randomly divided into two groups such as the A53T group and A53T + NBP group (*n* = 10, with a gender ratio of 1:1 in each group). The A53T group was administered with saline in the same manner as in the control group. The A53T + NBP group was intraperitoneally injected with NBP 0.2 ml daily for 14 days.

### Behavioral and Cognitive Assessments

#### Open Field Test

The subject mouse was placed in a white square container (40 × 40 cm^2^) with a camera installed at the top center of the box, and the behaviors of the subject mouse were recorded for 5 min. An automatic-recording tracking system was used to map the center and periphery zones, and the total moving distance of the subject mouse was calculated.

#### Rotarod Test

The rotarod test was performed using a rotarod treadmill (ZH-300B, Zhenghua). Mice were tested by a constant accelerating mode at 40 rpm/min from initial 10 rpm/min within a maximum recording time of 5 min. The retention time for the mouse to stay on a rotating rod was obtained from three repeated tests with an interval of 20 min. The mean retention time from the three trials was recorded.

#### Contextual Fear Conditioning

Each mouse was placed into a fear conditioning chamber (MED-VFC-NIR-M/R, MED Associates, United States) equipped with a video camera for a 20-min free exploration to establish baseline freezing behavior. On the first day of conditioning (day 1), each mouse was trained in the chamber for 10 min. The mouse was given a conditioned stimulus (a tone of 5 kHz and 70 dB for 30 s), followed by an unconditioned stimulus (an electric footshock of 0.5 mA for 1 s) that was delivered during the last 2 s of the conditioned stimulus. The conditioning was repeated five times during the 10 min, and the freezing behavior was scored. On the second day (day 2), the mice were returned to the same chamber respectively for 5 min to measure the freezing behavior in response to the context. Neither footshock nor tone was delivered. The freezing time during contextual memory testing was calculated. On the third day (day 3), the context of the chamber was altered significantly by changing the floor, walls, and background odor. The mice were placed into the chamber once again for 10 min. The conditioned stimulus was given to every mouse, which was repeated five times with an interval of 60 s. No footshock was delivered. The freezing time during cued memory testing was measured.

#### Olfactory Test

Each mouse was placed into a three-chamber box (100 cm × 60 cm × 20 cm), which was divided into two partitions with a hole at the bottom of each partition, wherein the mouse could run free across the chambers. The fresh padding was put into one of the lateral chambers, and the unchanged padding was put into another lateral chamber and the middle chamber, which was the same padding in the chamber wherein the mouse had been placed for 5 min at 48 h before the three-chamber test. The time for the mouse to stay on the unchanged padding was measured.

### Immunohistochemistry

#### Tissue Preparation

After the last saline or NBP injection, three mice were randomly selected from each group for the subsequent study. The brain tissue was perfused with saline followed by sodium pentobarbital anesthesia. Brain samples were fixed in 4% paraformaldehyde at 4°C for 24 h and dehydrated for 3 days until sinking to the bottom. A knife was used to cut the quick-frozen left brains (50-μm thick) of the mice, which passed through the corpus striatum, hippocampus, and substantia nigra. The sections were immersed in phosphate-buffered saline.

#### Tyrosine Hydroxylase (TH) Positive Cells and α-Synuclein Deposition in the Corpus Striatum and Substantia Nigra

The brain sections were incubated with primary antibodies, anti-TH (1:1,000, EP1532Y), and anti-α-synuclein (1:1,000), at room temperature overnight. After being rinsed with phosphate-buffered saline, the sections were incubated with secondary antibodies, antirabbit, and antirat IgG1 (1:1,000) at room temperature for 30 min. Subsequently, the sections were washed with phosphate-buffered saline for 5 min and stained with 3,3’-diaminobenzidine (DAB) solution for 30 s. Finally, the numbers of TH-positive cells in the nigra and striatum were counted under a microscope.

### Western Blotting Analysis

According to the manufacturer’s instructions, total protein was extracted from cytosolic and mitochondrial fractions using an ice-cold lysis buffer containing 50 mM Tris–HCl (pH 7.4), 1% Triton X-100, and 150 mM NaCl supplemented with a protease inhibitor cocktail (P8340, Merk). The protein concentrations were loaded onto 10% polyacrylamide gels, and then, the gels were transferred onto polyvinylidene difluoride membranes. The membranes were blocked with 5% skim milk for 1 h at room temperature and then incubated with primary antibodies overnight at 4°C. Thereafter, the membranes were incubated with secondary antibodies for 1 h at room temperature. Finally, the protein signals were detected by using a Tanon-5,500 Multi chemiluminescence gel imaging system. Band intensities were analyzed with Image J for Mac v1.52q.

### Transmission Electron Microscopy

The striatum was washed once in phosphate-buffered saline and then fixed with ice-cold 2.5% glutaraldehyde. The cell pellets were rinsed, dehydrated, and embedded sequentially. Finally, ultrathin sections were cut by using an ultramicrotome. The numbers of mitochondria and synapses were observed and measured under a JEM-1,400 electron microscope (JEOL Ltd., Japan).

### Statistical Analysis

Results were analyzed using Image J software and SPSS Statistics V 22.0 (SPSS, Inc., Chicago, IL, United States). Multiple group comparisons were performed using Student’s *t*-test or ANOVA with Tukey’s posttest. *P* < 0.05 was considered to indicate a statistically significant difference.

## Results

### NBP Alleviates Behavior Impairment and Cognitive Symptoms in A53T-α-Synuclein PD Mouse Model

To investigate the potential roles of NBP in the A53T-α-synuclein PD mouse model, a series of behavioral and cognitive assessments were performed, including open field test, rotarod test, olfactometry, and contextual fear conditioning.

**FIGURE 1:**
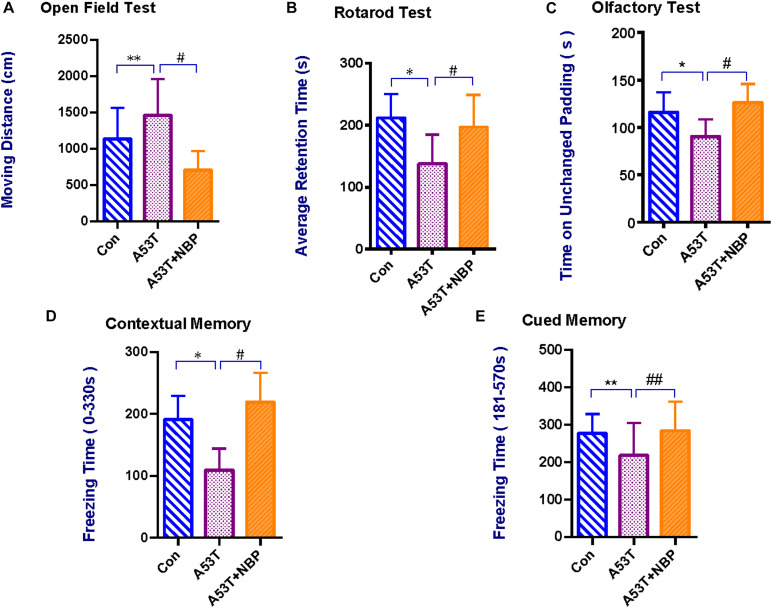
Behavioral and cognitive assessments. Compared with the control group, the A53T group exhibited a significant longer moving distance in the Open Field Test **(A)**, significant reductions in average retention time in the Rotarod Test **(B)**, time on unchanged padding in the Olfactory Test **(C)**, and freezing time during contextual memory and cued memory tests in Contextual Fear Conditioning **(D,E)** (**P < 0.01; *P < 0.05). After 2-week Dl-3-n-butylphthalide (NBP) treatment, the results were significantly reverted (^#^*P* < 0.05; ^##^*P* < 0.01). All data were expressed as mean ± SD.

*Open field test* was performed to assess anxiety-like behavior in PD mice (Asakawa et al., 2016). As shown in Figure 1A, the A53T group displayed a significant increase in moving distance compared with the control group. After NBP treatment, the moving distance was decreased significantly.

*Rotarod test* was further carried out to evaluate the coordination ability of the mice. As shown in Figure 1B, the mean retention time was significantly decreased in the A53T group than in the control group. After treatment of NBP, the mean retention time was prolonged significantly.

*Contextual fear conditioning* was performed to evaluate contextual memory (background stimuli continuously present in the apparatus in which tone–shock pairings occurred) and cued memory (a tone paired with footshock) in mice (Phillips and LeDoux, 1992). As shown in Figures 1D,E, compared with the control group, the freezing time during both contextual and cued memory tests in the A53T group was decreased significantly. After NBP treatment, the freezing time was prolonged.

An *olfactory test* was performed to assess the olfactory ability. As shown in Figure 1C, the time on the unchanged padding in the A53T group was less than that in the control group. After NBP treatment, the time on the unchanged padding was prolonged significantly.

Collectively, it was observed that the A53T-α-synuclein PD mice exhibited anxiety-like behavioral disturbance, impairment of coordination ability, memory deficits, and olfactory dysfunction. NBP could protect the mice from A53T-α-synuclein mutant-induced behavioral impairment and cognitive dysfunction.

### NBP Reactivates Dopaminergic Neurons in Corpus Striatum and Substantia Nigra of A53T-α-Synuclein PD Mouse Model

**FIGURE 2:**
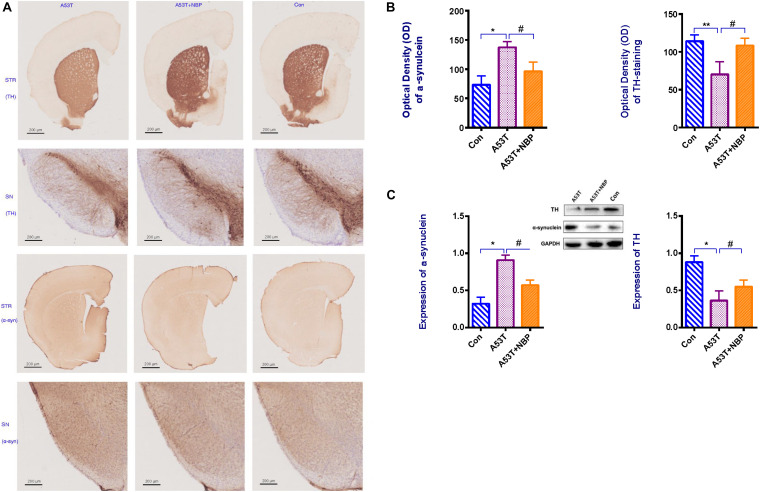
Tyrosine hydroxylase (TH) content and α-synuclein level. **(A)** Visualization of TH content and α-synuclein immunohistochemical staining in corpus striatum (STR) and substantia nigra (SN) of subject mice. Less TH content and higher level of α-synuclein were detectable in the A53T group. **(B,C)** When quantified, optical density (OD) and expression of α-synuclein were higher, and OD and expression of TH-positive cells were decreased in the A53T group (*P < 0.05; **P < 0.01). Following 2-week Dl-3-n-butylphthalide (NBP) application, the level of α-synuclein was lower and TH-positive cells were increased significantly (^#^*P* < 0.05). All data were expressed as mean ± SD.

To confirm and quantify the effects of NBP against the loss of dopaminergic neurons, Western blotting analysis, microscopy, and immunohistochemistry were performed to detect the number of tyrosine hydroxylase (TH)-positive cells in the corpus striatum and substantia nigra of the subject mouse. It was observed in Figure 2 that the number of TH-positive cells in the nigra and striatum was significantly decreased in the A53T group. After a 2-week NBP application, the number of TH-positive cells in the nigra and striatum recovered significantly, indicating that NBP could inhibit the loss of dopaminergic neurons.

### NBP Decreases α-Synuclein Accumulation in Corpus Striatum and Substantia Nigra of A53T-α-Synuclein PD Mouse Model

To confirm and quantify the effects of NBP against α-synuclein accumulation, the same experimental approach as detecting TH content was used to examine the level of α-synuclein in the corpus striatum and substantia nigra of the subject mouse. Compared with the control group, the level of α-synuclein in the brain tissue (Figure 2) was significantly higher in the A53T group. After a 2 week NBP application, the level was significantly lower, which indicated that NBP could against α-synuclein accumulation.

### NBP Recovers Synapses and Mitochondrial Morphology

**FIGURE 3:**
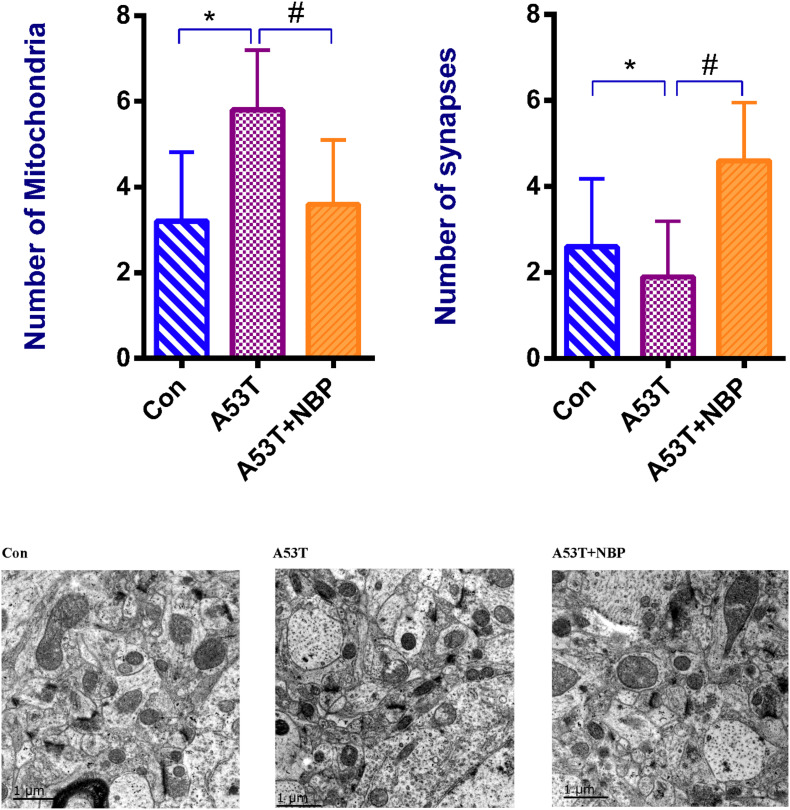
Mitochondrial morphology. Visualization of mitochondrial structural features. When quantified, the number of mitochondria was increased, and the number of synapses was decreased significantly in the A53T group (*P < 0.05). After Dl-3-n-butylphthalide (NBP) application, the number of mitochondria was reduced, and the number of synapses was increased significantly (^#^*P* < 0.05), while the mitochondrial structural features were partially recovered. All data were expressed as mean ± SD.

To explore the potential effects of NBP on mitochondria, the visualization of mitochondrial structural features was observed by electron microscopy. It is shown in Figure 3 that the number of synapses decreased, the number of mitochondria increased, and mitochondrial structural features were abnormal in the A53T group. After 2-week NBP treatment, compared with the control group, the number of mitochondria was significantly decreased, the number of synapses was increased, and mitochondrial morphology was partially recovered.

Taken together, these results indicated that the A53T-α-synuclein PD mouse model exhibited disturbance of synapses and mitochondrial morphology to a certain extent, and NBP might be capable of recovering the disturbance.

### NBP Rebalances the Mitochondrial Fission–Fusion and Inhibits Autophagy and Mitophagy

**FIGURE 4:**
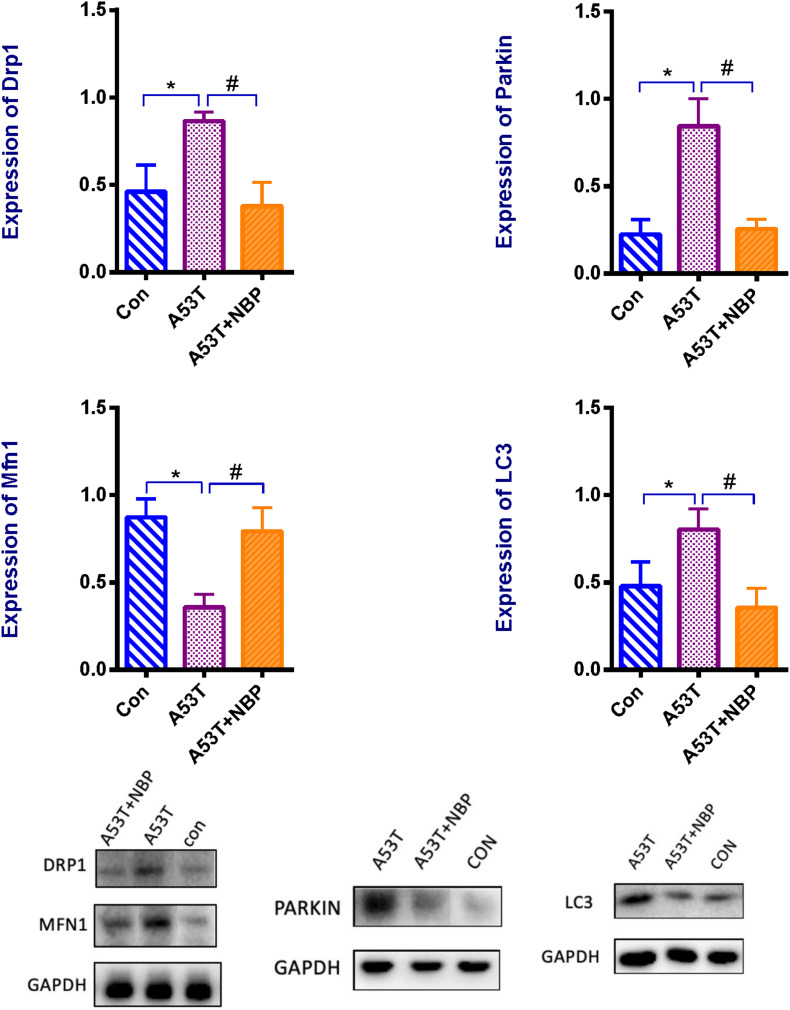
Mitochondrial proteins, autophagy, and mitophagy. Western blotting assay for the protein level of dynamin-related protein 1 (Drp1), and mitofusin 1 (Mfn1), Parkin, and LC3. When quantified, the expression of Drp1 was higher, and Mfn1 level was lower in the A53T group than that in the control group. Meanwhile, LC3 and Parkin levels were higher significantly. After 2-week NBP treatment, the results were all reversed significantly. All data were expressed as mean ± SD. *P < 0.05 and ^#^*P* < 0.05.

Mitochondrial morphology is highly regulated, and the regulation is mediated by mitochondrial dynamics including mitochondrial fission–fusion and mitophagy. To address the potential mechanisms of NBP by modulating mitochondrial dynamics in the A53T-α-synuclein PD mouse model, the expressions of mitochondrial fission protein (dynamin-related protein-1, Drp1) and mitochondrial fusion protein (mitofusin 1, Mfn1) and the indicators of autophagy (LC3) and mitophagy (Parkin) were detected by Western blotting analysis. As presented in Figure 4, the expression of Drp1 was higher, and the Mfn1 level was lower in the A53T group than that in the control group. Meanwhile, LC3 and Parkin levels were higher significantly. After 2-week NBP treatment, the results were all reversed significantly.

These data indicate that the A53T-α-synuclein PD mouse model exhibited multifaceted defects of mitochondrial dynamics. The modulation of mitochondrial fission–fusion and clearance was involved in the neuroprotective role of NBP against A53T-α-synuclein mutant-induced mitochondrial dysfunction.

## Discussion

The existence and detrimental effects of abnormal cytoplasmatic α-synuclein accumulation and aggregation and their aberrant forms in neurons and neuroglia are associated with neurodegenerative diseases, which are also called synucleinopathies. PD is one of the most common synucleinopathies. It is identified that the substitution of alanine with threonine at position 53 of the α-synuclein protein (A53T) is more prone to present an early onset PD and develop more severe manifestations (Bengoa-Vergniory et al., 2017). A53T α-synuclein variant is highly enriched in isolated mitochondria when compared to the wild-type protein or A30P α-synuclein variant (Devi et al., 2008). Some evidence from human and animal models of PD suggested that A53T α-synuclein might play a fundamental role in the control of neuronal mitochondrial dynamics such as mitochondrial fusion–fission, transport, and clearance (Pozo Devoto and Falzone, 2017; Lin et al., 2019). The mechanism that underlies the α-synuclein-mediated changes in mitochondrial dynamics is still not clear, but recent findings shed some light on the mitochondrial processes that regulate its size, distribution, and clearance.

NBP has been reported to process a potential therapeutic effect on neurodegenerative diseases indicated in some clinical and animal model studies (Huang et al., 2018; Chen et al., 2019; Luo et al., 2019). A recent study reports that NBP exerts neuroprotective effects against oxidative damage and mitochondrial dysfunction through its antioxidant property via an autophagy mechanism in a 1-methyl-4-phenylpyridinium + -induced cellular model of PD (Huang et al., 2010). Basing on these data, NBP may be valid in order to not only regulate mitochondrial dynamics but also to subsequently slow down PD progression, especially in familial cases or A53T α-synuclein variant.

The present study employed a human A53T α-synuclein transgenic PD mouse model, giving rise to dominant early onset PD and contributing to the mitochondrial defects (Pozo Devoto and Falzone, 2017). It examined whether NBP can also exhibit neuroprotection in this PD model and further investigated the possible effects on the maintenance of mitochondrial dynamics.

Behavioral and cognitive assessments are crucial to evaluating whether a medicine exhibits protective effects on dopaminergic neurons, while the number of TH-positive cells is one of the most important pathological indicators for assessing whether a drug exhibits neuroprotective effects on dopaminergic neurons. In the present study, the A53T-α-synuclein PD mouse model exhibited the basic characteristics of PD such as anxiety-like behavioral disturbance, impairment of coordination ability, memory deficits, and olfactory dysfunction, and the main hallmarks such as loss of dopaminergic neurons and α-synuclein accumulation in the corpus striatum and substantia nigra. All these data are consistent with the findings in previous studies for α-synuclein A53T mutant PD mouse.

After 2 week administration of NBP, using our well-characterized induced human α-synuclein A53T mutant transgenic PD mouse model, it was observed that the detected results of indicators were reversed significantly. Collectively, the data indicate that NBP plays favorable roles in the improvement of behavior impairment and cognitive symptoms, protective effect on dopaminergic neurons, and reduction in α-synuclein deposition for early onset familial PD. By investigating the effect of NBP on the disturbance of mitochondrial homeostasis induced by A53T-α-synuclein mutant, we are likely to uncover a novel finding that sheds light on the mitochondrial processes that regulate mitochondrial fission–fusion and mitophagy.

The maintenance of mitochondrial homeostasis involves modulating mitochondrial dynamics, which includes balancing the mitochondrial fission–fusion, regulating mitochondrial morphology, and inhibiting autophagy and mitophagy (Youle and Narendra, 2011; Pernas and Scorrano, 2016; Vasileiou et al., 2019). In 1976, it was reported for the first time that PD is associated with mitochondria. Some evidence suggests that mitochondrial dysfunction plays an important role in PD progression and studies have highlighted the key role of α-synuclein aggregation in influencing mitochondrial homeostasis (Pozo Devoto and Falzone, 2017; Lin et al., 2019; Hijaz and Volpicelli-Daley, 2020; Park et al., 2020). Mitochondria morphology is highly regulated by a dynamic balance between mitochondrial fusion and fission (Burté et al., 2015). Mitochondria fusion can reduce the occurrence of mitophagy by protecting the functional proteins and non-damaged mitochondrial DNA from dysfunctional mitochondria. Mfn1, a protein from the dynamin-related GTPase family, can modulate the mitochondrial membrane fusion at the mitochondrial outer membrane (Itoh et al., 2013). Fission is the opposite of fusion, and the fission process can decrease the mitochondrial size and enhance mitochondrial axonal transport and mitophagy. Drp1 is one of the main proteins involved in the fission process (Itoh et al., 2013). Mitophagy is a specific autophagy process aimed at recycling mitochondria in lysosomes, which is mediated by the microtubule-associated proteins 1A/1B light chain 3A (LC3) in the autophagosome. As a component of the ubiquitin–proteasome system that is a major pathway in α-synuclein degradation (Jêśko et al., 2017), Parkin is involved in the maintenance of mitochondrial metabolism, which is particularly critical in the richly arborized dopaminergic neurons of nigra and plays a key role in the removal of defective mitochondria via mitophagy (Geisler et al., 2010; Chen et al., 2018; Giguère et al., 2018; Jêśko et al., 2019). Parkin and LC3 are the PD-related proteins participating in the regulation of mitophagy, which can selectively degrade mitochondria via autophagy to clear damaged mitochondria (Vasileiou et al., 2019).

Manipulating mitochondrial fusion and fission has been considered a potential mitochondrial therapy in recent years (Wang et al., 2016; Bido et al., 2017). In the present study, Western blotting analysis showed that compared with the control group, Drp1, LC3, and Parkin levels were increased, and Mfn1 level was decreased in the A53T group; the microscopic observation indicated that the number of mitochondria in the striatum was decreased, and mitochondrial structural features were abnormal in the A53T group. Collectively, all these data indicated the negative interaction of the A53T α-synuclein mutant and the mitochondrial processes that regulate its size, distribution, and clearance. Fortunately, after NBP treatment, all these results were reversed, and the microscopy showed that the mitochondrial morphology was partially recovered. In light of this, it is tempting to speculate that a function of NBP is to play potential roles in the manipulation of the mitochondrial fission and fusion via blocking Drp1 GTPase activity and promoting Mfn1 level, and orchestrating mitophagy via inhibiting Parkin and LC3 levels.

## Perspective and Future Outlook

Despite the predominant localization in the cytosol and nucleus of neurons, it is found that α-synuclein is localized in mitochondria in the nigra of a PD postmortem brain. Specifically, it has been reported that α-synuclein is localized in the three main mitochondrial structures including the mitochondrial matrix and the outer and inner mitochondrial membranes, where α-synuclein interacts with these mitochondrial components. Compared with the wild-type protein or A30P variant, the A53T α-synuclein variant is more highly enriched in isolated mitochondria, and the inner mitochondrial membrane has a high α-synuclein concentration determined by immunoelectron microscopy and Western blotting analysis (Devi et al., 2008; Devi and Anandatheerthavarada, 2010). However, the exact localization of α-synuclein within mitochondria remains unclear. Mitochondria are highly dynamic organelles undergoing coordinated cycles of fission and fusion, referred to as “mitochondrial dynamics,” in order to maintain their shape, distribution, and size. Mitochondrial fusion and fission are crucial events, and it is evident that these dynamic morphological transitions control cell fate decisions (Tilokani et al., 2018). The present study highlighted a novel neuroprotective effect of NBP on the maintenance of mitochondrial dynamics in the A53T α-synuclein PD mouse model. In the future, further experiments would shed light on the precise mechanisms of NBP on mitochondrial homeostasis, elucidating how these events are regulated, not only from a molecular but also biological point of view.

## Conclusion

In the present study, the A53T-α-synuclein PD mouse model exhibited pathological characteristics of PD and indicated that mitochondrial fission–fusion and clearance may be involved. Furthermore, the present study highlighted a valuable neuropharmacological role of NBP, due to its ability to modulate mitochondrial dynamics in the A53T-α-synuclein PD mouse model. Further studies and a more in-depth understanding of this mechanism is required to understand NBP neuroprotection and may lead to the identification of new therapeutic opportunities. In the near future, more experiments based on the novel insight should be performed to shed light on the precise mechanisms of NBP on mitochondrial homeostasis induced by α-synuclein mutation.

## Data Availability Statement

The original contributions presented in the study are included in the article/supplementary material, further inquiries can be directed to the corresponding author/s.

## Ethics Statement

The animal study was reviewed and approved by the Animal Care and Use Committee of Institute of Laboratory Animal Science, Chinese Academy of Medical Sciences.

## Author Contributions

HYL and YFL conceived and designed the study, completed the measurements and data analysis, and wrote the manuscript. HQW performed the experiments and contributed to the interpretation of data. LZ and MSW were responsible for the animal studies, data collection, and all statistical analysis. YFL conceived the study, obtained funding for the study, and assisted with manuscript preparation. All authors read and approved the final manuscript.

## Conflict of Interest

The authors declare that the research was conducted in the absence of any commercial or financial relationships that could be construed as a potential conflict of interest.
